# Ureteroileal bypass: a new technic to treat ureteroenteric strictures in urinary diversion

**DOI:** 10.1590/S1677-5538.IBJU.2017.0014

**Published:** 2018

**Authors:** Guilherme P. Padovani, Marcos F. Mello, Rafael F. Coelho, Leonardo L. Borges, Adriano Nesrallah, Miguel Srougi, William C. Nahas

**Affiliations:** 1 Divisão de Urologia, Faculdade de Medicina da Universidade de São Paulo, SP, Brasil

**Keywords:** Urinary Diversion, Cystectomy, Urinary Bladder

## Abstract

**Objective:**

To present our technique of ureteroileal bypass to treat uretero-enteric stric- tures in urinary diversion.

**Materials and Methods:**

One hundred and forty-one medical records were reviewed from patients submitted to radical cystectomy to treat muscle-invasive bladder cancer between 2013 and 2015. Twelve (8.5%) patients developed uretero-enteric anastomotic stricture during follow-up. Five patients were treated with endoscopic dilatation and double J placement. Four were treated surgically with standard terminal-lateral im- plantation. Three patients with uretero-enteric anastomotic stricture were treated at our institution by “ureteroileal bypass”, one of them was treated with robotic surgery.

**Results:**

All patients had the diagnosis of uretero-enteric anastomotic stricture via computerized tomography and DTPA renal scan. Time between cystectomy and diag- nosis of uretero-enteric anastomotic stricture varied from five months to three years. Mean operative time was 120±17.9 minutes (98 to 142 min) and hospital stay was 3.3±0.62 days (3 to 4 days). Mean follow-up was 24±39.5 months (6 to 72 months). During follow-up, all patients were asymptomatic and presented improvement in ure-terohydronephrosis. Serum creatinine of all patients had been stable.

**Conclusions:**

Latero-lateral ureter re-implantation is feasible by open or even robotic surgery with positive results, reasonable operation time, and without complications.

## INTRODUCTION

Bladder cancer is the ninth most frequently diagnosed cancer worldwide, with the highest incidence rates observed in men in Southern and Western Europe and North America (1). Approximately one third of patients diagnosed with bladder cancer have muscle invasive disease (MIBC). The standard definitive treatment for MIBC is radical cystectomy (RC) with pelvic node dissection and urinary reconstruction (2, 3).

Complications of RC and diversion can appear after months or years of surgical treatment. Most complications can be managed conservatively, but some of the late adverse events require surgical treatment. Uretero-enteric strictures are a late complication after cystectomy and diversion that occur in 2% to 15% of patients (4–6). Multiple treatment alternatives have been proposed to those strictures with variable success rates. Ureteral reimplantation is still considered the gold standard surgical treatment (7). However, the surgical approach to the uretero-enteric anastomosis can be challenging due to fibrosis and adhesions. We propose herein a technical modification aiming to minimize ureteral dissection; the technique involves a latero-lateral anastomosis of the dilated ureter with the ileal conduit without detaching the ureter from the intestinal segment. Our experience with this technical modification is described.

## MATERIALS AND METHODS

Twelve (8.5%) patients developed uretero-enteric anastomotic stricture among 141 patients submitted to radical cystectomy to treat MIBC between 2013 and 2015 in our institution. Bricker's procedure is used in external ileal conduit diversion, and, Studer's technique, in ileal neobladder. In both diversions, the ureter is reimplanted separated with 2 continuous running sutures.

Follow-up after radical cystectomy was (mean: 13.2 months, 3-38 months). Patients were evaluated 3 months after surgery, and 6 months successively, with CT scan and serum creatinine; if there was any progressive hydronephrosis, an increase in serum creatinine, lumbar pain, or pye-lonephritis, a DTPA renal scan was evaluated.

### None of these patients were treated with radiotherapy

In all of them a percutaneous procedure was attempted as treatment, but was possible in only five patients, since they weren't completely stenotic. Seven patients were treated surgically; four of them were treated with a resection of the stenotic area and a standard terminal-lateral im-plantation. The last three patients in this series were treated by a “latero-lateral anastomosis” and were the objective of this publication. Before surgery, the technique was explained to patients, and informed consent was applied. There was no selection of patients for this technique.

### Technique of latero-lateral ureter re-implantation

**Figure 1:**
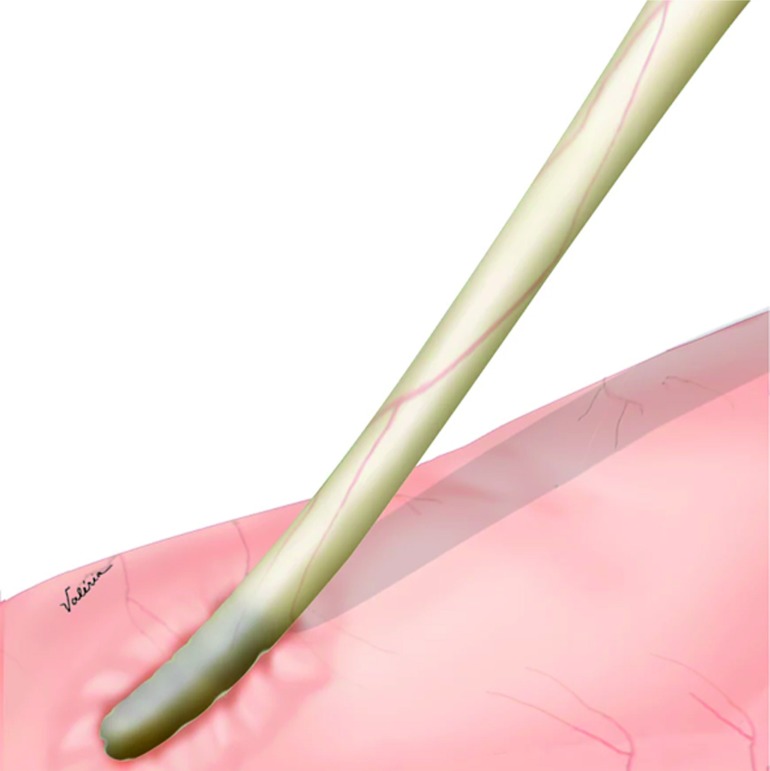
Ureter mobilization.

The surgery begins with the lysis of adhesions, and the identification of diversion. The stenotic area at the anastomosis site is identified without any prior catheter placement, and left in place without excision. The proximal portion of the dilated ureter is dissected, and mobilized; (Figure-1).

**Figure 2:**
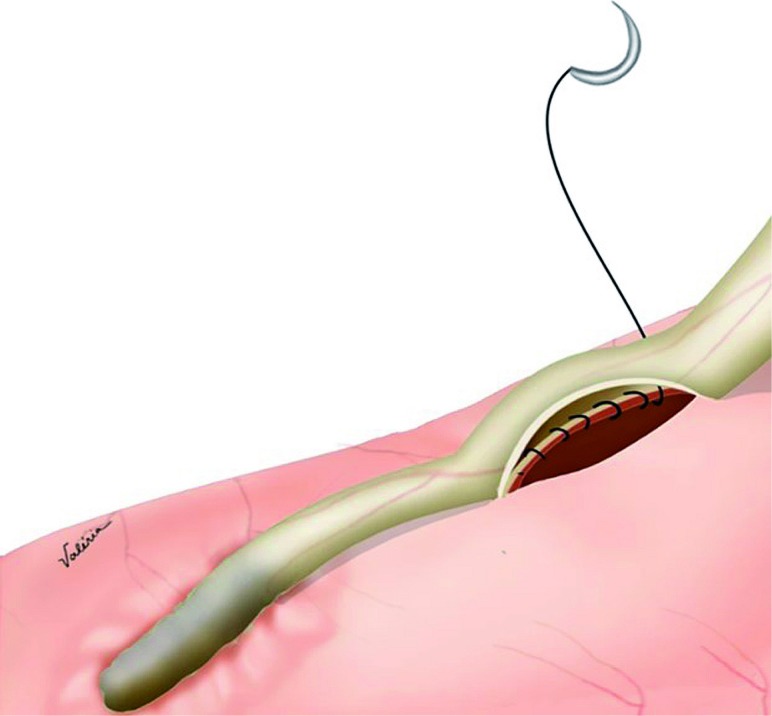
Anastomosis in urinary diversion.

**Figure 3:**
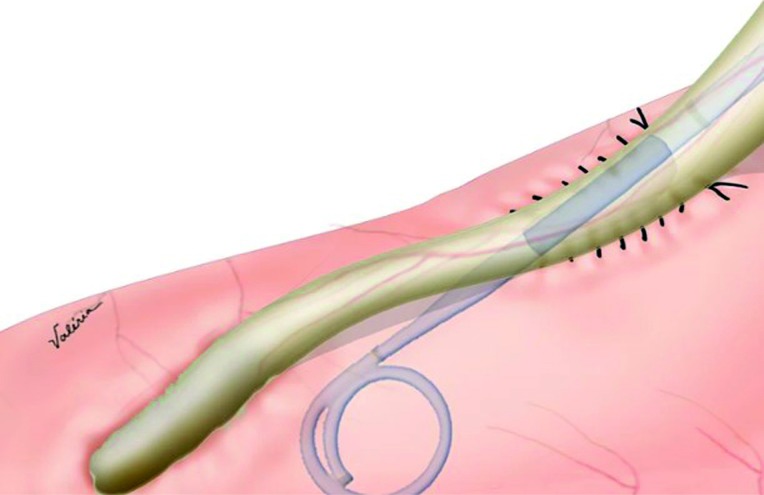
Ureter-ileal bypass with 2J catheter.

A 1cm incision is performed in the lateral wall of the ureter and at the urinary diversion; a latero-lateral anastomosis is performed with 2 continuous 5-0 polyglactin sutures (Figure-2). The posterior side of anastomosis is made first, then a double J catheter 6x26 is placed (Figure-3), and finally the anterior side is sutured. A peritoneal drain is placed. The double J catheter is removed after 4 weeks of the surgery.

### RESULTS

Two patients had ileal conduits and one had an orthotopic ileal neobladder done by Studer's technique as an urinary reconstruction after cystectomy. Two patients were symptomatic (flank pain and pyelonephritis) and one was asymptomatic. All patients had the diagnosis of uretero-enteric anastomotic stricture with grade 3 hydronephrosys (8) on the computed tomography scan (CT), without any image of cancer recurrence, and obstructive curves on DTPA renal scan. One renal unit was affected in each patient: two at the right and one at the left side.

Time between cystectomy and the diagnosis of stenosis was five months to three years (3 years in the first case, 18 months in the second and 5 months in the third case).

Mean operative time was 120±17.9 minutes (98 to 142 min) and hospital stay was 3.3±0.62 days (3 to 4 days). There weren't any post-operative complications (Clavien-Dindo Classification (9)) in these patients. Mean follow-up was 29.3 months (10 to 48 months).

During follow-up, patients were evaluated 3 months after surgery, and 6 months successively, with CT scan and serum creatinine. All of them were asymptomatic and had improvement in the ureterohydronephrosis. Two cases still had minor ureterohydronephrosis, and were submitted to DTPA, which proved that there was no obstruction. One patient had completely resolved ureterohydronephrosis and no further exams were necessary. During follow-up, all patients were asymptomatic. Serum creatinine of all patients was stable (Table-1).

**Table 1:** Study Group Carachteristics.

AA	Cr (mg/dL)	Urinary	Cr (mg/dL)	Symptoms	Side	Cr (mg/dL)	Follow-up
	pre-cistectomy	Diversion	post-cistectomy			post-re-implatation	(months)
				
67 years	0.69	OIN	1.0	Flank pain	R	0.9	48
75 years	0.60	IC	1.6	Pyelonephritis	R	1.1	30
70 years	0.88	IC	1.34	None	L	1.2	9

**AA =** age at analysis; **OIN =** orthotropic ileal neobladder; **IC =** Ileal Conduit; **R =** Right; **L =** Left

### Comments

The uretero-enteric anastomotic stricture occurs commonly within the first 1-2 years pos-toperatively, regardless of the type of implant (10, 11). The incidence is cumulative over time, and stenosis has been reported up to 6 years postoperatively (12).

Patients can be asymptomatic, present with an insidious onset, or be detected by an increased serum creatinine or from regular follow-up imaging exams. Some patients present flank pain. Pyelonephritis is usually a late manifestation that accompanies long-standing obstruction with grade 4 nephropathy and renal parenchymal loss (13–18).

Even with the upper tract dilation, the diagnosis may be challenging. Frequently a dynamic imaging exam is needed, such as a CT urography, DTPA renal scan, anterograde nephrostogram, or loopogram/neobladder cystography. Most commonly the left ureteral implantation is affected, due to a wider mobilization of the ureter (17, 18).

The upper tract dilation may be a result of recurrent malignancy. Certainly, all cases of obs-truction must be rigorously investigated to rule out ureteral luminal recurrence or retroperitoneal metastatic spread of the disease. This can be done using CT and magnetic resonance image, and in suspicious cases of cancer recurrence, cytology or even biopsy are necessary (18, 19).

But the most common cause is a stenotic process at the ureteric reimplantation site due to an ischemic condition as a consequence of ureteric dissection or after radiation (18–21).

Recently, due to advances in invasive radiology and endourology percutaneous imaging guided procedures, endoscopic techniques have been an option in treatment (22). The referred success rate widely varied; balloon dilation (16%-83%), (23–30); endoureterotomy (30%-50%), (26) and/or stent placement (45%) (31).

Laparoscopic or even open surgery after cystectomy can be challenging due to the presence of intra-abdominal adhesions and modified anatomy after reconstruction (4). However, the standard management is an open surgical revision with excision of the strictures ureteral segment and wide reanastomosis. The reported success rates are between 76% and 93% in 3 years, with acceptable morbidity (5, 19).

Benign ureteral strictures are a result of an ischemic insult and the key to their prevention and management is meticulous surgical technique. During its repair, pouch mobilization and identification of the stenotic area may be a difficult procedure.

The rationale of a latero-lateral anastomosis is to perform a minimal ureteric dissection and mobilization to avoid compromising even more its blood supply (32). The stricture is not excised and a proximal latero-lateral anastomosis is easily created between the dilated ureter and the bowel segment. The procedure may be performed by an open approach or by a minimally laparoscopic/robotic approach as described in our series.

This is an initial series with few cases, which precludes the statistical comparison of results with any other series, such as with a series of cases in which they were reimplanted with the standard technique. The safety of the new technique shown in the initial cases allows its use in the following cases, and soon we will have a series for comparison.

## CONCLUSIONS

Latero-lateral uretero-enteric anastomosis is a feasible treatment option for benign anastomotic strictures with encouraging midterm outcomes. It can be performed either by open or by minimally invasive approaches with good perioperative outcomes.
